# Performance of Multiparametric Models in Patients With Brugada Syndrome: A Systematic Review and Meta-Analysis

**DOI:** 10.3389/fcvm.2022.859771

**Published:** 2022-04-14

**Authors:** Hui-ting Wei, Wei Liu, Yue-Rong Ma, Shi Chen

**Affiliations:** ^1^School of Basic Medicine, Chengdu University of Traditional Chinese Medicine, Chengdu, China; ^2^West China School of Public Health, Sichuan University, Chengdu, China; ^3^Department of Cardiology, West China Hospital, Sichuan University, Chengdu, China

**Keywords:** Brugada syndrome, multiparametric models, predictive performance, sudden cardiac death (SCD), implantable cardioverter-defibrillator (ICD)

## Abstract

**Background:**

Multiparametric models have shown better risk stratification in Brugada syndrome. Recently, these models have been validated in different populations.

**Aims:**

To perform a systematic review and meta-analysis of the predictive performance of three validated multiparametric models (Delise model, Sieria model, and Shanghai score).

**Methods:**

We searched PubMed, Embase, MEDLINE, Web of Science, and Ovid for studies validating the risk multiparametric model. A Sieria score > 2 and Shanghai score ≥ 4 were considered to indicate higher risk. Performance estimates were summarized using a random-effects model.

**Results:**

Seven studies were included, with sample sizes of 111–1,613. The follow-up duration was 3.3–10.18 years. The Sieria model had a pooled area under the curve (AUC), sensitivity, and specificity of 0.71 [95% confidence interval (CI): 0.67–0.75], 57% (95% CI: 35–76), and 71% (95% CI: 62–79), respectively. The Shanghai score had an AUC of 0.63–0.71, 68.97–90.67% sensitivity, and 43.53–63.43% specificity. The AUC of the Delise model was 0.77–0.87; however, the optimal cut-off was not identified.

**Conclusions:**

The three models exhibited moderate discriminatory ability for Brugada syndrome. The Sieria model has poor sensitivity and moderate specificity, whereas the Shanghai score has poor specificity and moderate sensitivity.

## Introduction

Brugada syndrome (BrS) is an inherited heart disease with a typical electrocardiogram (ECG) pattern that carries a risk of ventricular arrhythmias (VAs) and sudden cardiac death (SCD) (1, 2), with initial symptoms ranging from syncope to SCD (3). Currently, implantable cardioverter-defibrillators (ICD) are the only effective treatment for VAs in BrS, but are strongly associated with immediate and long-term risks, are expensive and require long-term intervention, and may reduce the patient's quality of life. Therefore, accurate risk stratification is of critical importance in managing BrS (4).

To date, BrS risk stratification remains challenging (5, 6). Several studies have focused on predicting ventricular fibrillation (VF) occurrence in BrS. Spontaneous type 1 ECG (Sp1) (7), history of syncope caused by VA (8), and family history of SCD (9) are all predictive factors for high-risk patients, but their prognostic power in BrS patients is limited. Some studies that have incorporated multiple predictive factors may be helpful in the risk stratification of BrS patients. Delise et al. (10) reported that patients with a basal type 1 ECG and syncope, family history of SCD, and positive electrophysiological study (EPS) had higher risk of VAs. Sieria et al. (11) reported that comprehensive evaluation of Sp1, sudden cardiac arrest, history of syncope, early familial sudden cardiac arrest, inducible EPS, and sinus node dysfunction are important for stratifying VF risk in BrS patients. The Shanghai score, which includes the presence of Sp1, type 1 ECG due to fever or medication, clinical history of arrhythmia or arrhythmic syncope, family history, and genetic test result, has predictive value for BrS patients (12, 13).

These models have been validated in a small sample size (14) and more recently in independent cohorts (15). To understand the strength and quality of the available evidence of these prognostic multiparametric models, we performed a systematic review and meta-analysis of all relevant articles to summarize the prognostic and discriminatory performance of these models in BrS patients.

## Methods

### Database Search

This study was conducted in accordance with Preferred Reporting Items for Systematic Reviews and Meta-Analyses (PRISMA) statement-related procedures. Several multiparametric models have been proposed but have not been validated in other cohorts (16, 17). Therefore, we performed a systematic search for published studies evaluating multiparametric models [Delise model (10), Sieria model (11), and Shanghai score (12)]. The brief introduction of Delise model, Sieria model, and Shanghai score models is in Supplementary Table S1. We searched PubMed, Embase, MEDLINE, Web of Science, and Ovid for studies published up to January 6, 2022. The full search strategies are described in Supplemental Material. Abstracts, editorials, letters, case reports, and review papers were excluded from this study. Research manuscripts for which the full text was unavailable or with missing information were also excluded. Only complete analytical studies published in peer-reviewed journals were included.

### Study Selection and Data Extraction

Two authors (H.T.W. and W.L.) performed duplicate literature screening based on the titles and abstracts and subsequently screened the records retrieved from the search independently. Then, they performed a full-text review of the screening records. If there was disagreement during this period, a third reviewer (S.C.) joined the discussion and resolved it by consensus. The two investigators extracted the following information independently: general patient information (age, sex, region); number of patients in the study; follow-up duration; number of SCD or SCD-equivalent cases. The outcomes analyzed were SCD, appropriate defibrillator therapy as determined through ICD interrogations and/or ventricular tachycardia/VF by ECG during follow-up. For the meta-analysis, the data for true and false positives and true and false negatives were extracted directly from the article or from data reported in the article, and the summary results were compiled in a 2 × 2 table.

### Risk of Bias Assessment

Two investigators (H.T.W. and W.L.) evaluated the risk of bias of the included studies independently using the Newcastle–Ottawa Scale (18). The scale consists of a list of eight items involving patient selection, cohort comparability, and outcome assessment. If there was disagreement between the two investigators, a third reviewer (S.C.) participated in the discussion and negotiated a solution.

### Data Analysis

Dichotomous variables are expressed as proportions (percentages), and continuous variables are expressed as the mean ± SD or median (range). Meta-analyses were performed using a random-effects model, the odds ratios (ORs), C-statistics or area under the curve (AUC) with the 95% CI of each study were weighted according to their size. Pooled estimates of sensitivity, specificity, positive predictive value, the positive likelihood ratio (PLR) and negative likelihood ratio (NLR) were pooled using a random-effects model with the using Mantel-Haenszel methods based on inverse variance weighting. Sensitivity analysis used the leave-one-out approach to assess whether the pooled results were influenced by a single study. According to the different types, subgroup meta-analyses are performed by pooling the studies into 2 subgroups: prospective study and retrospective study. A *p*-value < 0.05 and *I*^2^ > 50% are considered to indicate statistical significance. The statistical analyses were performed using RevMan 5.4 and Stata 17.

## Results

### Study Selection

**Figure 1:**
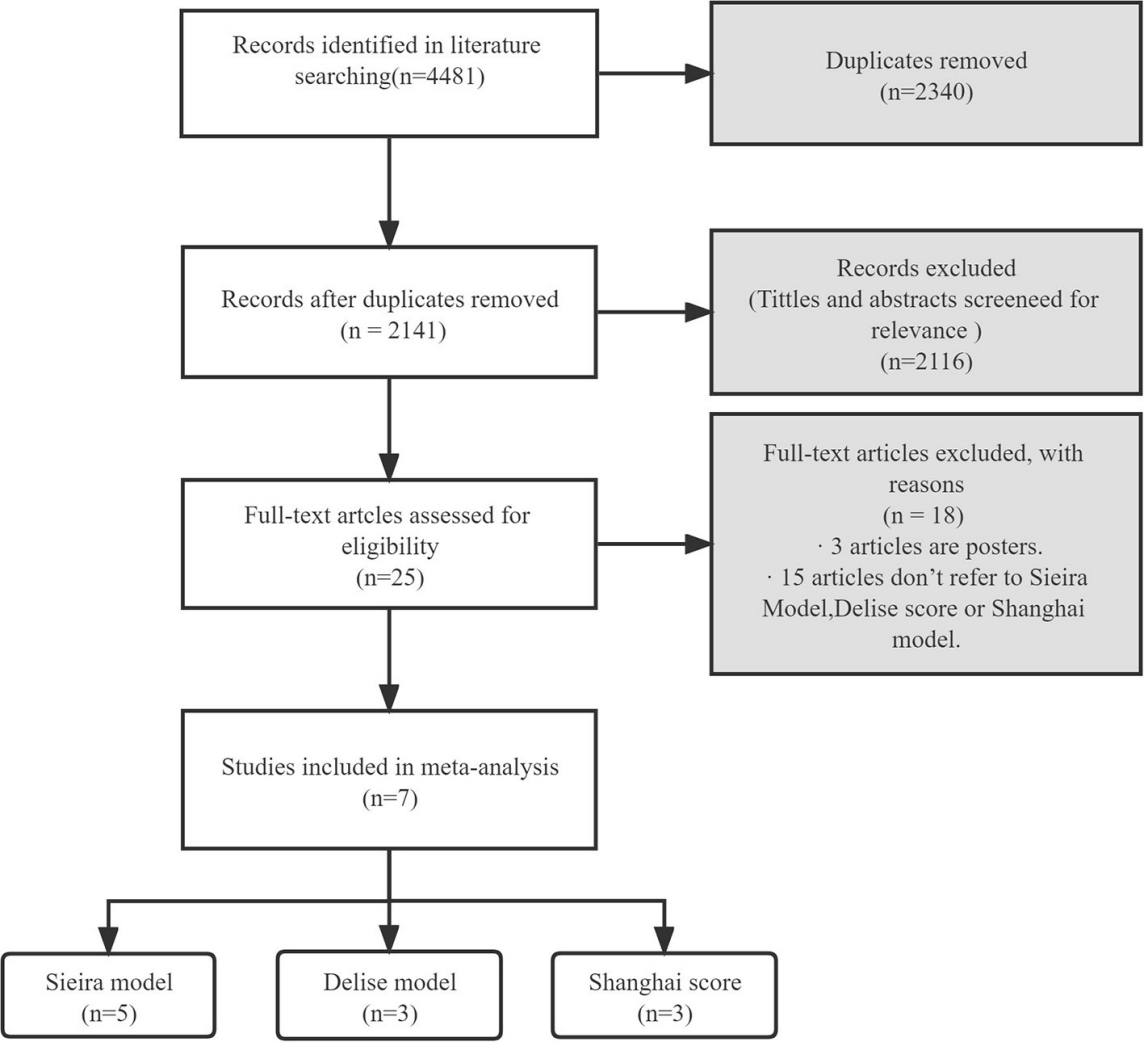
Flowchart describing the publication search and selection algorithm.

Figure 1 shows the flow chart of the literature search strategy. The search identified 4,481 citations. Seven observational studies (three prospective and four retrospective) were included, of which four were multicenter. Table 1 presents the characteristics of the seven studies included in the systemic review and meta-analysis. The studies had sample sizes of 111–1,613. The mean patient age was 41.1–47.1 years, and male patients comprised 58.3–95.2% of the sample. The follow-up duration was 3.3–10.18 years. All studies considered a composite arrhythmic endpoint formed by SCD and/or aborted cardiac arrest, ongoing VAs, and/or appropriate ICD therapies. Five studies reported the prognostic performance of the Sieria model, three studies reported the Shanghai score, and three studies reported the Delise model. The methodological quality of the studies based on the Newcastle–Ottawa Scale score is reported in Table 2. The seven studies were graded as good quality.

**Table 1:** Baseline study characteristics of multiparameter-risk-prediction model validation studies in Brugada syndrome.

**References**	**Study design**	**Model**	**Region**	**Samples**	**Age, y**	**Sex (male %)**	**Time of follow-up**	**SCD (%)**
Delise et al. (10)	Prospective, five centers	Delise model	Italy	320	43 (33–54)	258 (81)	40 months (IQ20-67)	17 (5.3%)
Sieira et al. (11)	Retrospective, single-center	Sieira model	Belgium	400	41.1 ± 17.8	233 (58.3)	80.7 months ± 57.2	34 (8.5%)
								
Kawada et al. (12)	Retrospective, single-center	Shanghai score	Japan	393	44.5 (36–56)	374 (95.2)	97.3 months (39.7 to 142.1)	43 (10.9%)
Letsas et al. (14)	Prospective, single-center	Sieira model/Delise model	Greek	111	45.3 ± 13.3	86 (77.4)	4.6 years ± 3.5	7 (6.3%)
Probst et al. (15)	Prospective, 15 centers	Sieira model/Shanghai score	France	1613	44 ± 13	356 (77)	9.4 years ± 4.1	27 (5.9%)
Rodríguez-Mañero et al. (19)	Retrospective, 24 centers	Sieira model/Shanghai score/Delise model	Spain	831	42.8 ± 13.1	561 (77)	10.18 years ± 4.77	47 (5.7%)
Chow et al. (20)	Retrospective, 2 centers	Sieira model	United Kingdom	192	47.1	112 (58.3)	5.1 years ± 2.76	22 (11.4%)

**Table 2:** Study quality was assessed using the Newcastle–Ottawa Scale.

**References**	**Selection**	**Comparability**	**Outcome**	**Total score**
Delise et al. (10)	3	1	3	7
Sieira et al. (11)	4	1	3	8
Kawada et al. (12)	3	1	3	7
Letsas et al. (14)	3	1	3	7
Probst et al. (15)	3	1	3	7
Rodríguez-Mañero et al. (19)	3	2	2	7
Chow et al. (20)	3	1	3	7

### Sieria Model

**Figure 2:**
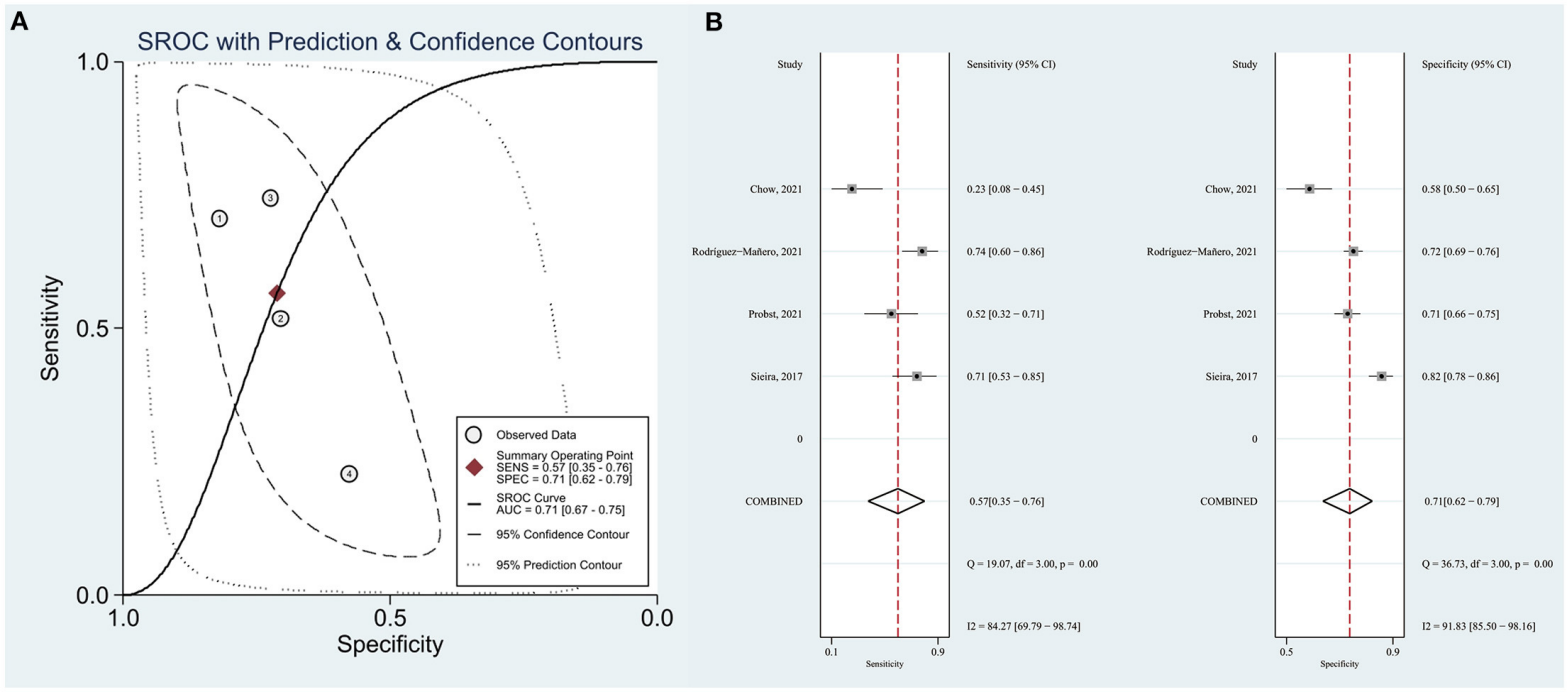
**(A)** SROC in the Sieria model. **(B)** Forest plots of sensitivity and specificity.

**Figure 3:**
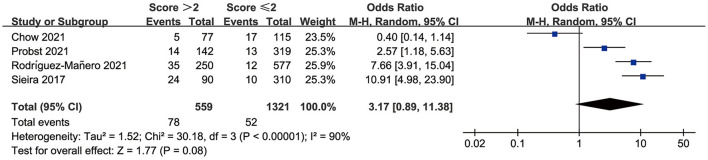
Meta-analysis results of ORs in the Sieria model.

Sieira et al. (11) proposed that patients with a score > 2 points showed significantly higher event probability. Therefore, we evaluated the resolution of the Sieria model using a score of 2 as the cut-off point. The area under the summary receiver operating characteristic (SROC) curve (AUC) was 0.71 (95% CI: 0.67–0.75) (Figure 2A). The pooled sensitivity, specificity, PLR and NLR were 57% (95% CI: 35–76), 71% (95% CI: 62–79), 3.12 (0.81–11.48) and 0.30 (0.09–1.05), respectively (Figure 2B). The pooled OR of Sieira score > 2 points for the prediction of arrhythmic events are reported in Figure 3. Letsas et al. (14) showed that the Sieria model had an AUC of 0.87 (95% CI 0.75–0.99, *P* = 0.001), 100% sensitivity, and 51.9% specificity without a defined cut-point.

**Figure 4:**
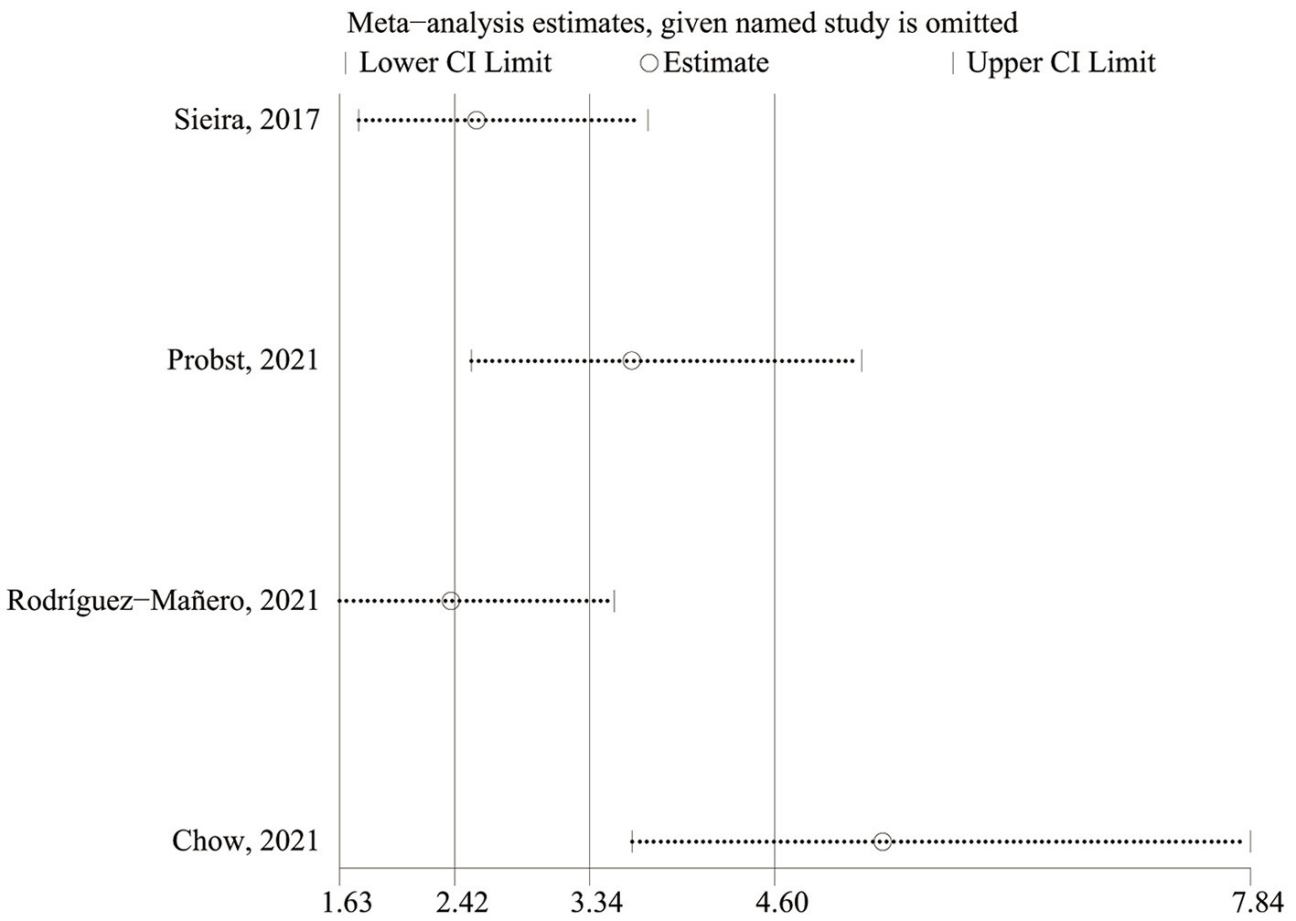
Sensitivity analysis of the extracted C-statistics.

There was a high level of heterogeneity for the OR between the studies (*I*^2^ = 90.0%; *P* < 0.0001). A one-study-removed approach in the sensitivity analysis was used to assess whether any of the studies would alter the overall results (Figure 4). After the study by Chow et al. (20) had been removed, the sensitivity analysis showed significantly reduced heterogeneity for OR. In the two prospective studies, the presence of Sieria score > 2 points was associated with an increased risk for ventricular arrhythmias and SCD (OR: 8.79; 95% CI: 5.26–14.69; *p* < 0.001), heterogeneity was not significant. When the studies were retrospective, the pooled OR was 1.19 [95% confidence interval (CI): 0.66–2.14], heterogeneity was high, see in Supplementary Figure S1.

### Shanghai Score

**Figure 5:**
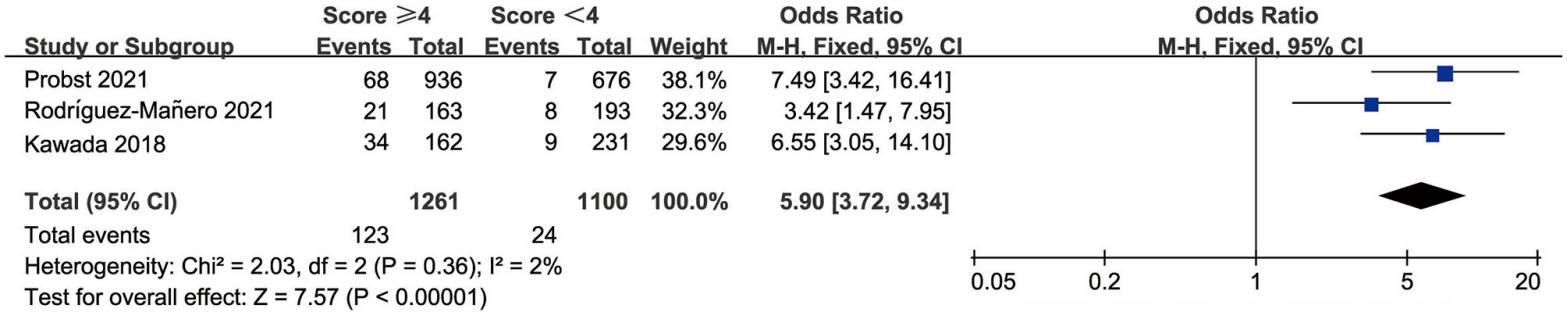
Meta-analysis results of ORs in the Shanghai score.

The Shanghai score defines people with a score ≥ 4 as high to highest risk. We evaluated Shanghai score discrimination when 4 points was used as the cut-off point. Three studies reported data for the prognostic performance of the Shanghai score. The AUC was 0.626–0.712, sensitivity was 68.97–90.67%, and specificity was 43.53–63.43% (Table 3). The pooled OR was 5.90 (95% CI: 3.72–9.34) and there was no significant heterogeneity (Figure 5).

**Table 3:** The AUC, sensitivity and specificity of Shanghai score in the different studies.

**Study**	**Shanghai score < 4**	**Shanghai score ≥ 4**	**AUC**	**Sensitivity**	**Specificity**
Probst et al. (15)	676/7	936/68	0.671 (0.647–0.694)	90.67 (81.7–96.2)	43.53 (41.0–46.0)
Rodríguez-Mañero et al. (19)	193/8	163/21	0.626 (0.574–0.677)	68.97 (49.2–84.7)	56.27 (50.7–61.7)
Kawada et al. (12)	231/9	162/34	0.712 (0.665–0.757)	79.07 (64.0–90.0)	63.43 (58.1–68.5)

### Delise Model

Three studies reported the prognostic accuracy of the Delise model, for which the optimal cut-off was not given. Therefore, pooled analysis could not be performed. Delise et al. (10) incorporated syncope, basal type 1 ECG, family history of SCD, and positive EPS as a risk model in 245 BrS patients who underwent EPS, and the risk model yielded a C-statistic of 0.87 (95% CI: 0.82–0.90). Validation by Rodríguez-Mañero et al. (19) showed that this model had an AUC of 0.77 in a total of 831 patients. The Delise model yielded an AUC of 0.87 (95% CI 0.73–1.00; *P* = 0.002), validated by Letsas et al. (14) in 111 patients, and 71.4% sensitivity and 86.5% specificity.

## Discussion

In this meta-analysis of a multiparametric model for prognostic performance in BrS, we determined that binary assessment of a Sieria score > 2 and a Shanghai score ≥ 4, considered higher risk, demonstrated moderate capability for predicting an arrhythmic event in BrS patients. The Sieria model had low sensitivity and moderate specificity; the Shanghai score had moderate sensitivity and lower specificity.

Sieria et al. (11) studied 400 BrS patients via a multiparametric approach that included ECG patterns, early familial SCD antecedents, induced electrophysiology, SCD as manifested by syncope or miscarriage, and sinus node dysfunction, reporting a predictive performance of 0.82 in a single-center consecutive cohort and 0.81 in a validation cohort. Consistent with their findings, validation by Rodríguez-Mañero et al. (19) demonstrated that the AUC was 0.81. In addition, Letsas et al. (14) showed that the Sieria model had an AUC of 0.87. However, validation by Chow et al. (20) demonstrated that in 192 patients from two tertiary institutions in the UK, the Sieria model had an AUC of 0.58. Two important factors generate low prognostic performance: temporal variation in cohort characteristics, and invasive EPS with a total of five possible points is less common in the UK. None of the above studies clearly indicated the AUC cut-off value. Therefore, it is unclear which point can be defined as high-risk. We used 2 points as the cut-off value proposed by Sieira et al. (11) and determined that the predictive performance was moderate, with an AUC of 0.71 (95% CI: 0.62–0.75), whereas the sensitivity was low, with a pooled sensitivity of 57% (95% CI: 35–76). Several factors may be responsible for this, such as the Sieira cohort originating from a single center, and the validation study demonstrating that the proportion of family history of SCD was lower than that of the Sieira cohort.

The Shanghai score for diagnosing BrS was calculated based on several risk factors, and the scoring system also had prognostic value (AUC = 0.759, 71.2% sensitivity, 73.1% specificity) (12). Event-free survival curves showed that patients with 3.5 points had moderate risk for VAs while patients with 4–5 points were at high risk, and patients with >5.5 points were in the highest risk category. Recently, Probst et al. (15) evaluated Shanghai BrS accuracy and reported a predictive power AUC of 0.73 (95% CI: 0.67–0.79). Rodríguez-Mañero et al. (19) reported that the score performance was 0.80 and also did not define an AUC cut-off value. We obtained moderate prognostic accuracy for the Shanghai score because of lower specificity when 3.5 points was used as the cut-off point. A meta-analysis demonstrated that inducible VAs were predictive of arrhythmic events in asymptomatic BrS patients (21) and EPS proved to be a relevant prognostic indicator. This lower specificity may be explained by the Shanghai score without the need for an EPS.

Delise et al. (10) evaluated the risk factors for VA/SCD in a primary prevention BrS cohort, where the presence of a basal type 1 ECG was combined with other clinical risk factors, including syncope, family history of SCD, and positive EPS. No events occurred in patients without any or only one of these risk factors. Letsas et al. (14) assessed the predictive power of the risk scores in 111 consecutive patients and reported that the predictive capacity demonstrated an AUC of 0.87. More recently, Rodríguez-Mañero et al. (19) evaluated the accuracy of the Delise model and estimated that the predictive capacity from the AUC was 0.77. Unlike the Sieria model or the Shanghai score, we were unable to obtain the AUC, sensitivity, and specificity of the Delise model for a given number of risk factors. The predictive power of this risk model for evaluating arrhythmic risk remains uncertain when BrS patients have multiple risk factors. This casts doubt on the utility of this score model for the primary SCD prevention. The Sieria model incorporated the spontaneous type-1 ECG, early familial SCD, positive EPS, syncope, SND and aborted SCD, all the variables had high risk factors. The Shanghai Score System which included electrocardiographic recordings, genetic results, clinical characteristics, and family history was used to diagnosis of BrS, only some of the items were reported to be associated with arrhythmic events alone. So, the Sieria model had higher specificity and lower sensitivity compared with Shanghai Score System.

### Limitations

First, we could not include all studies in the meta-analysis due to limitations such as publication in a non-English language. Second, we did not obtain original data from the authors and based our analysis only on the data reported in the studies. Third, the studies we included were predominantly retrospective, and bias and confounding might have been present.

## Conclusions

The three models exhibited moderate discriminatory ability for BrS. The Sieria model has poor sensitivity and moderate specificity, whereas the Shanghai score has poor specificity and moderate sensitivity.

## Data Availability Statement

The raw data supporting the conclusions of this article will be made available by the authors, without undue reservation.

## Author Contributions

All authors listed have made a substantial, direct, and intellectual contribution to the work and approved it for publication.

## Conflict of Interest

The authors declare that the research was conducted in the absence of any commercial or financial relationships that could be construed as a potential conflict of interest.

## Publisher's Note

All claims expressed in this article are solely those of the authors and do not necessarily represent those of their affiliated organizations, or those of the publisher, the editors and the reviewers. Any product that may be evaluated in this article, or claim that may be made by its manufacturer, is not guaranteed or endorsed by the publisher.
