# Supplementary material for: Performance of Multiparametric Models in Patients With Brugada Syndrome: A Systematic Review and Meta-Analysis
Source: Front Cardiovasc Med. 2022 Apr 14;9:859771. doi: 10.3389/fcvm.2022.859771 (PMC9047913; doi:10.3389/fcvm.2022.859771)

Supplementary - Database search algorithms

The search algorithms uesd in this study are presented below. All database were searched from January 1st 2011 until January 6th 2022. The number of retrieved items is presented next to each database.

Search for PubMed (n=1080):

((brugada) OR (brugada syndrome)) AND((prognosis) OR (risk assessment) OR (predictive value) OR (outcome)), 2011-01-01~2022-01-06

Search for Embase (n=1421):

(brugada or brugada syndrome).af. And (prognosis or risk assessment or predictive value or outcome).af. limit 3 to (human and english language and yr="2011 - Current")

Search for Medline (n=874):

(brugada or brugada syndrome).af. And (prognosis or risk assessment or predictive value or outcome).af. (english language and humans and yr="2011 -Current")

Search for Web of Science (n=1106):

(TS=(brugada OR brugada syndrome)) AND TS=(prognosis OR risk assessment OR predictive value OR outcome), 2011-01-01~2022-01-06

Supplementary Table S1 : Individual prognostic variables in the 3 risk models.

**Model**	**Risk factor**		**Relative value**	**Categories**
Delise Score	Spontaneous type 1 ECG		-	Individuals at higher risk are those with a baseline type 1 ECG pattern who have at least 2 of the following risk factors:<br>- Syncope<br>- Family history of SCD<br>- Positive EPS
	Syncope		-	
	Family history of SCD		-	
	Positive EPS		-	
Sieira Score	Pontaneous type 1 ECG		1	A score > 2 showed a significantly higher event probability
	SCA		4	
	Syncope		2	
	Early familial SCA < 35yo		1	
	Inducible EPS		2	
	SND		3	
Shanghai Score	. ECG (12-lead/ambulatory)	Spontaneous type 1 ECG at nominal or high leads	3.5	- Moderate risk: 3.5<br>- High risk: 4 to 5<br>- Highest risk ≥5.5
		Fever-induced type 1 ECG at nominal or high leads	3	
		Type 2 or 3 ECG pattern that converts with drug provocation challenge	2	
	Clinical history	Unexplained cardiac arrest or documented VF/polymorphic VT	3	

	Nocturnal agonal respirations	2
	Suspected arrhythmic syncope	2
	Syncope of unclear mechanism/unclear etiology	1
	Atrial flutter/fibrillation in patients < 30 y without alternative etiology	0.5
Family history	First- or second-degree relative with definite BrS	2
	Suspicious SCD (fever, nocturnal, Brugada aggravating drugs) in a first- or second-degree relative	1
	Unexplained SCD at	0.5
Genetic test result	Probable pathogenic mutation in BrS susceptibility gene	0.5

Supplementary Figure S1 : Subgroup analysis of Sieira model between prospective and retrospective studies.

Study or Subgroup	Score >2 Events	Score >2 Total	Score ≤2 Events	Score ≤2 Total	Weight	Odds Ratio M-H, Fixed, 95% CI
**9.1.2 Prospective Study**						
Rodríguez-Mañero 2021	35	250	12	577	21.1%	7.66 [3.91, 15.04]
Sieira 2017	24	90	10	310	11.2%	10.91 [4.98, 23.90]
**Subtotal (95% CI)**		**340**		**887**	**32.3%**	**8.79 [5.26, 14.69]**
Total events	59		22			
Heterogeneity: Chi² = 0.45, df = 1 (P = 0.50); I² = 0%						
Test for overall effect: Z = 8.29 (P < 0.00001)						
**9.1.3 Retrospective Study**						
Chow 2021	5	77	17	115	43.2%	0.40 [0.14, 1.14]
Probst 2021	14	142	13	319	24.5%	2.57 [1.18, 5.63]
**Subtotal (95% CI)**		**219**		**434**	**67.7%**	**1.19 [0.66, 2.14]**
Total events	19		30			
Heterogeneity: Chi² = 7.94, df = 1 (P = 0.005); I² = 87%						
Test for overall effect: Z = 0.57 (P = 0.57)						
**Total (95% CI)**		**559**		**1321**	**100.0%**	**3.64 [2.54, 5.22]**
Total events	78		52			
Heterogeneity: Chi² = 30.18, df = 3 (P < 0.00001); I² = 90%						
Test for overall effect: Z = 7.04 (P < 0.00001)						
Test for subgroup differences: Chi² = 25.21, df = 1 (P < 0.00001), I² = 96.0%						

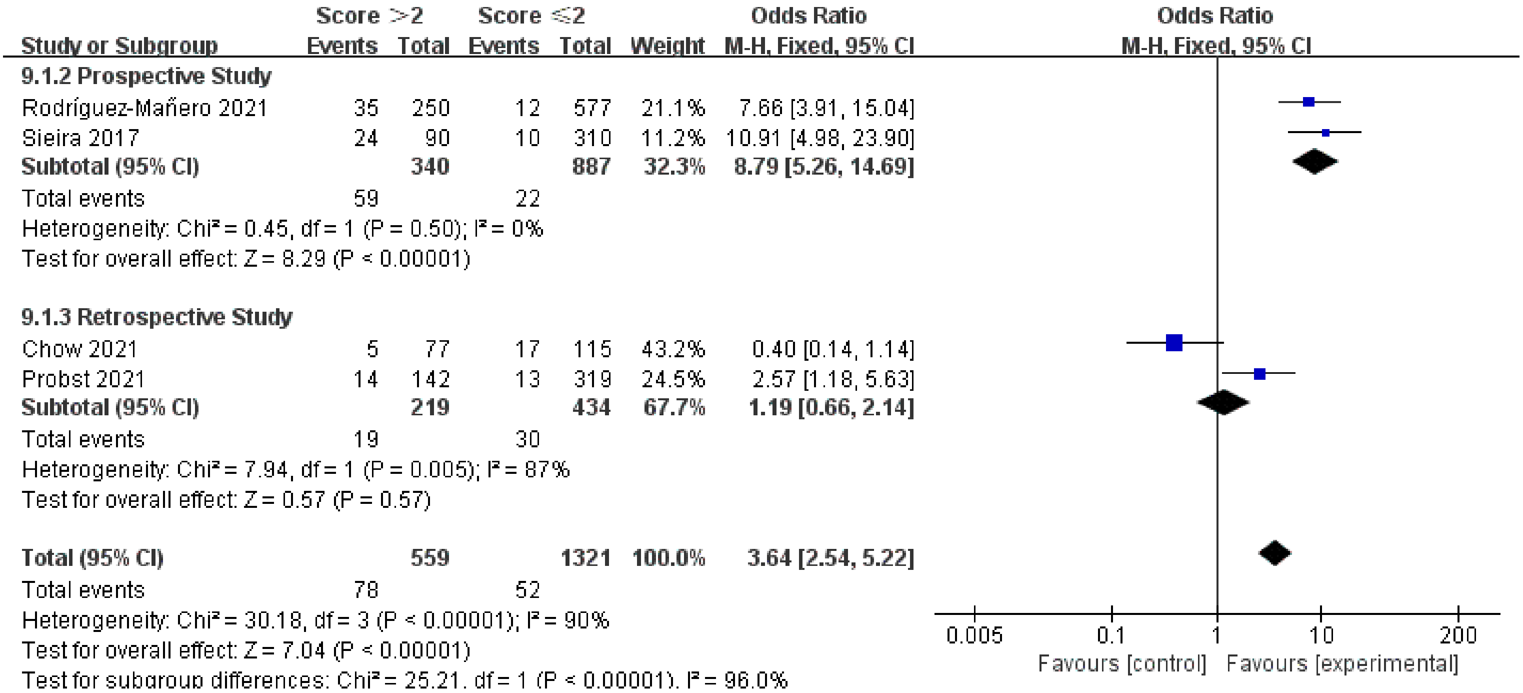

Supplement: Supplementary file 1 [file Data_Sheet_1.pdf]
